# Understanding Repression Under Secretion Stress in *Trichoderma reesei* During Cellulase Expression

**DOI:** 10.3390/microorganisms14061371

**Published:** 2026-06-21

**Authors:** Reshma Jadhav, Güler Demirbas Uzel, Julien Charest, Igor Nikolaev, Sharief Barends, Robert Ludwig Mach, Astrid Rosa Mach-Aigner

**Affiliations:** 1 Institute of Chemical, Environmental and Bioscience Engineering, TU Wien, Gumpendorfer Str. 1a, 1060 Vienna, Austria; reshma.jadhav@tuwien.ac.at (R.J.); julien.charest@tuwien.ac.at (J.C.); robert.mach@tuwien.ac.at (R.L.M.); 2 Christian Doppler Laboratory for Optimized Expression of Carbohydrate-Active Enzymes, Institute of Chemical, Environmental and Bioscience Engineering, TU Wien, Gumpendorfer Str. 1a, 1060 Vienna, Austria; gueler.demirbas@tuwien.ac.at; 3 IFF Health & Biosciences, Willem Einthovenstraat 4, 2342BH Oegstgeest, The Netherlands; igor.nikolaev@iff.com (I.N.); sharief.barends@iff.com (S.B.)

**Keywords:** *Trichoderma reesei*, cellulases, unfolded protein response, repression under secretion stress

## Abstract

The filamentous fungus *Trichoderma reesei* is one of the most important workhorses for industrial enzyme production, but the cellular mechanisms that balance protein folding stress with secretion, such as the unfolded protein response (UPR) and repression under secretion stress (RESS), are still not fully understood. In this study, we set out to clarify how these pathways contribute to secretion in both laboratory settings and industrial-scale fermentations. Exposure to the reductive agent dithiothreitol for 5 h increased transcript levels of UPR-related genes at least 6-fold, and, simultaneously, transcript levels of target genes *cbh1* and *egl2* were reduced at least 5- or 6-fold, respectively. Interestingly, RESS was detected even when UPR was suppressed by the prevention of protein *de novo* synthesis, pointing to a non-hierarchical relation of the two mechanisms. With the aim to understand on which levels RESS is acting, in particular, whether it is transcription initiation or transcript stability, an experiment involving blocking the transcription was performed. Further, a recombinant strain with an exchanged promoter had an at least 45-fold-increased *cbh1* transcript level, while a terminator exchange did not increase *chb1* transcript levels, indicating that RESS operates mainly at the level of transcription initiation. Importantly, whole transcriptome data from industrial cellulase production did not reveal the signatures of UPR or RESS despite the heavy secretory load. Instead, expression profiles highlighted the induction of diverse hydrolytic enzymes and pathway adjustments that support efficient production.

## 1. Introduction

*Trichoderma reesei*, a filamentous fungus, is a cornerstone organism in industrial biotechnology due to its extraordinary capacity to secrete large quantities of cellulases, hemicellulases, and other enzymes essential for breaking down lignocellulosic biomass into simple sugars that can be used in the production of biofuels and chemicals such as ethanol. Additional biotechnological applications of these enzymes include improving food digestibility, enhancing brewery fermentation, facilitating the cold extraction of olive oil, and serving as a host for recombinant protein production. Its role as a plant symbiont and its potential in biocontrol further extend its versatility in agriculture [1,2,3,4].

The unfolded protein response (UPR) is a highly conserved eukaryotic cellular stress response activated by the accumulation of unfolded or misfolded proteins in the endoplasmic reticulum (ER) [5]. The UPR aims to restore ER homeostasis by attenuating general protein translation, upregulating molecular chaperones, enhancing protein folding capacities, and promoting the degradation of faulty proteins [6,7]. In mammals, yeast, and many fungi, UPR signaling is mediated by key effectors such as IRE1, PERK, and ATF6, and its sustained activation can lead to apoptosis if the cell fails to rebalance protein folding loads [5,8]. Repression under secretion stress (RESS) is another cellular feedback mechanism that downregulates the transcription of genes encoding secreted proteins during ER stress to lessen the burden on the folding machinery [9].

In filamentous fungi, including *T. reesei*, it is reported that UPR plays a central role in handling the stress resulting from high-level secretion of enzymes in industrial fermentation processes. Excessive production of (hemi-)cellulases or heterologous proteins in overexpressing strains can trigger ER stress, leading to activation of UPR, which modulates secretion machinery, and RESS, which transiently represses the transcription of genes for secreted proteins [10,11]. Unlike *Saccharomyces cerevisiae*, which lacks a clear RESS mechanism [12], filamentous fungi usually display RESS prominently when exposed to folding and glycosylation inhibitors such as dithiothreitol (DTT) and tunicamycin [13,14,15].

Recent transcriptome analyses indicate that regulatory network components and cultivation conditions, such as carbon source (e.g., lactose or xylan), critically influence the degree and targets of RESS and UPR activation in *T. reesei* [6,16]. The mechanistic link between UPR and RESS in *T. reesei* is increasingly apparent as stress-induced downregulation of cellulase, and hemicellulase gene transcription is tightly coordinated with ER stress markers, molecular chaperone expression, and folding capacity. For example, the disruption of endoplasmic reticulum-associated protein degradation (ERAD) pathway components (*hrd1*, *hrd3*, *der1*) not only compromises ER homeostasis and fungal growth under stress, but also triggers the UPR response and RESS-mediated repression of cellulase genes, especially *bgl1*, reducing β-glucosidase production [17]. This dynamic feedback ensures that accumulation of misfolded proteins in production settings is counterbalanced by a reduction in production pressure, maintaining secretion efficiency and cell viability [7,18].

Producing hydrolytic enzymes such as cellulases on an industrial scale can indeed induce the UPR. This is due to the exceptionally high secretory load placed on the ER during industrial processes, particularly when organisms like *T. reesei* are engineered or cultivated to overproduce these enzymes [6,9,19]. Environmental and growth stresses, such as suboptimal oxygen transfer, elevated temperatures, or the presence of chemical stressors (e.g., DTT to mimic folding stress) [19] as well as substrate and carbon source shifts can result in the surge of enzyme production, further increasing ER stress [20].

*T. reesei* is indispensable in industrial biotechnology for enzyme production. However, the potential interplay between the UPR and RESS mechanisms remains poorly understood and, therefore, continues to be a target of research and production optimization. Accordingly, this study addressed several key questions, including whether and how UPR and RESS are interconnected in *T. reesei*. It also investigated whether UPR and RESS can be suppressed by preventing *de novo* protein synthesis. Furthermore, the underlying mechanism of RESS was examined: specifically, whether it involves mRNA degradation and/or repression at the level of transcription initiation. Finally, the occurrence of UPR and RESS was studied for both standard laboratory conditions and industrial production settings.

## 2. Materials and Methods

### 2.1. Fungal Strains

*T. reesei* RL-P37 (a publicly available mutant strain derived from NG-14 by UV radiation; intact Cre1-mediated carbon catabolite repression), RL-P37_Δ*pyr4* (bearing a point mutation in *pyr4*; uridine auxotroph; provided by IFF Nutrition & Biosciences (Oegstgeest, The Netherlands)) and the industry strain GEN-3A (derived from RL-P37; intact Cre1-mediated carbon catabolite repression; provided by IFF Nutrition & Biosciences (Oegstgeest, The Netherlands)) were maintained at 30 °C either on potato dextrose agar (PDA) or malt extract (MEX) agar, with 5 mM uridine for the *pyr4* negative strain. The prototrophic recombinant strains RL-P37 p*gpd1*::*cbh1*::t*gpd1*::*pyr4* (termed RL-P37_T1; this study), RL-P37 p*cbh1*::*cbh1*::t*gpd1*::*pyr4* (termed RL-P37_T2; this study) and RL-P37 p*gpd1*::*cbh1*::t*cbh2*::*pyr4* (termed RL-P37_T3; this study) were kept on Mandels-Andreotti (MA) medium [21] containing 1% glucose without peptone.

### 2.2. Growth Conditions

*T. reesei* RL-P37 and the recombinant strains were subjected to replacement experiments. For this, mycelia were pre-cultured in 1 L Erlenmeyer flasks on a rotary shaker (180 rpm) at 30 °C for 24 h in 250 mL of MA medium supplemented with 1% (*v*/*v*) glycerol as the sole carbon source (exponential growth phase). A total of 10^9^ conidia/liter (final concentration) was used as inoculum. Pre-grown mycelia were washed and equal amounts were resuspended in 20 mL production medium containing the cellulase-inducing compound sophorose (provided by IFF Nutrition & Biosciences (Oegstgeest, The Netherlands) [22]) either with 10 mM DTT as UPR inducer or without DTT as a non-UPR condition. DTT was used in earlier studies in a concentration range from 1 to 20 mM; however, no impairment in growth nor a changed expression of housekeeping genes were observed, which allows us to exclude cytotoxic effects [23,24,25]. In total, 10 μM cycloheximide (CHX) was added as a protein translation inhibitor to study the reversal of RESS [25]. In total, 16 μg/mL 5,6-dichloro-1-beta-D-ribofuranosylbenzimidazole (DRB) was added as a Pol II elongation inhibitor [26] and the flasks were incubated for 3, 5 and 8 h at 30 °C with 180 rpm. Mycelia (in steady-state phase) were harvested using Miracloth (Calbiochem, San Diego, CA, USA) at respective timepoints and stored in liquid nitrogen until further processed for RNA extraction followed by gene expression analysis.

*T. reesei* GEN-3A was cultivated in three biological replicates in fed-batch fermentation as described in [27]. Briefly, either cellulase producing conditions were applied, i.e., cultivation on minimal medium with glucose for a period of 24–36 h to build up biomass (exponential growth phase) followed by a glucose–sophorose feed for another 120–150 h, or, as a control condition, cultivation on minimal medium with glucose. The pH and temperature were maintained constant at 5.5 and 28 °C. Samples (in steady-state phase) were collected at 24 h intervals at the indicated time points, flash-frozen in liquid nitrogen and stored at −80 °C until RNA extraction.

### 2.3. Isolation of Chromosomal DNA and Genotype Verification

Chromosomal DNA was extracted from mycelium by phenol-chloroform extraction [28]. RNA was degraded using RNase A (Thermo Scientific, Waltham, MA, USA). DNA was precipitated with isopropanol, washed with 70% ethanol, and dissolved in ddH_2_O. For testing the genotype, 10 ng chromosomal DNA was used as the template in a 25 μL PCR mixture using Taq DNA Polymerase (New England Biolabs, Ipswich, MA, USA) according to the manufacturer’s instructions. All primers used are listed in Table 1. A Quick-Load^®^ 1 kb DNA Ladder (New England Biolabs) was applied for the estimation of the fragment size in agarose gel electrophoresis and DNA sequencing was performed at Microsynth (Balgach, Switzerland).

### 2.4. Plasmid Construction and Purification

**Figure 1:**
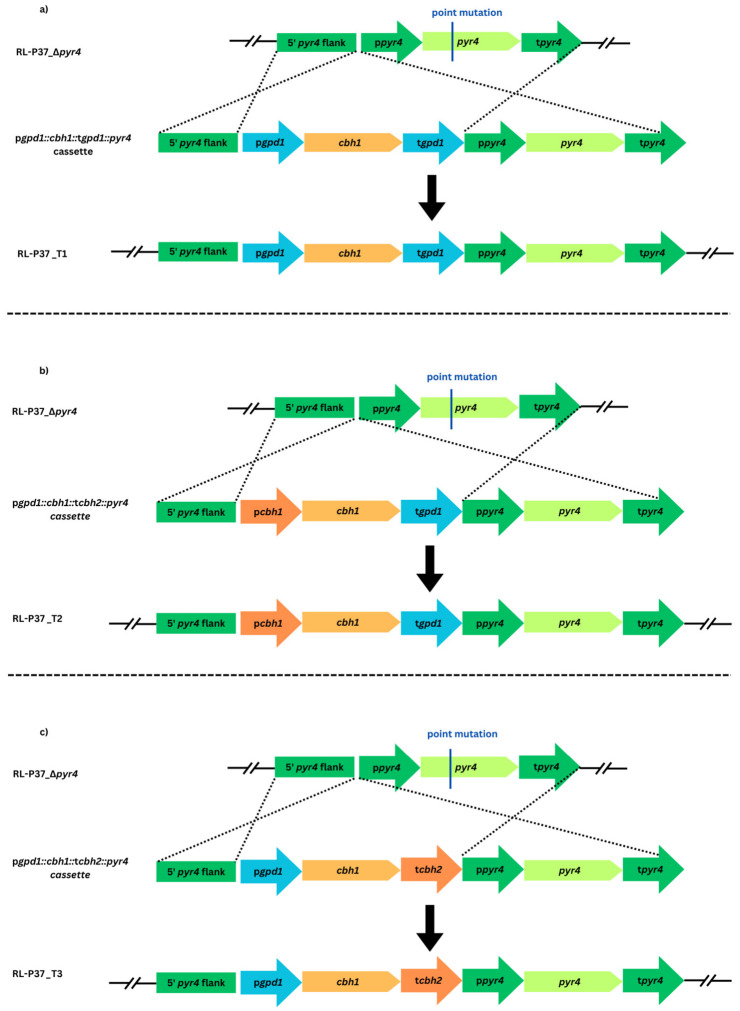
Modification of the *pyr4* locus during strain generation. Schematic representation of homologous recombination between the *pyr4* locus of the auxotrophic recipient strain (RL-P37_Δ*pyr4*) and a template cassette bearing the fragments of interest. The strategies to obtain the recombinant strains RL-P37_T1 (**a**), RL-P37_T2 (**b**) and RL-P37_T3 (**c**) are presented. The *pyr4* flanking regions are presented as dark green boxes, the *pyr4* gene as a light green arrow, the *pyr4* promoter and terminator as dark green arrows, the *gpd1* promoter and terminator as blue arrows, the *cbh1* promoter and terminator as dark orange arrows and the *cbh1* gene as a light orange arrow. Regions for double crossover recombination during fungal transformation are indicated by black dotted crossed lines.

The template from which the fragments were amplified are from genomic DNA of RL-P37 (p*gpd1*, t*gpd1*, *cbh1*) and pRPyr4-pc1g [29]. A PCR of the fragments was performed with Q5^®^ High-Fidelity DNA Polymerase followed by cloning in *E. coli* cells (Top10), according to manufacturing’s instructions. To construct the RL-P37_T1 strain, the promoter of the *gpd1* gene (p*gpd1*) and the terminator of *gpd1* (t*gpd1*) were added before and after the *cbh1* gene, resulting in the plasmid p*gpd1*::*cbh1*::t*gpd1*::*pyr4* (Figure 1a). Similarly, to construct the RL-P37_T2 strain, the t*gpd1* was fused to the *cbh1* promoter and gene to generate the plasmid p*cbh1*::*cbh1*::t*gpd1*::*pyr4* (Figure 1b). Finally, to construct the RL-P37_T3 strain, the p*gpd1* was incorporated before the *cbh1* gene and the terminator from the *cbh2* gene (t*cbh2*) to generate the plasmid p*gpd1*::*cbh1*::t*cbh2*::*pyr4* (Figure 1c). The list of primers used in this study is given in Table 1.

Plasmid preparation was performed using the Miraprep protocol [30]. To purify the plasmid 0.1 volume of 3 M sodium acetate, 2.5–3 volume ice-cold 100% ethanol was added to the isolated plasmid. The mixture was then vortexed thoroughly and incubated at −80 °C for 1 h or overnight. The mixture was centrifuged at 13,000 rpm, 4 °C for 30 min. The pellet was washed twice with 0.5 mL ice-cold 75% ethanol followed by centrifugation for 10 min, each time. Decant and even the trace amount of ethanol was removed using pipette. The air-dried pellet was resuspended in appropriate volume of nuclease-free water or TE buffer.

### 2.5. Generation of T. reesei Protoplasts

Half of the RL-P37_Δ*pyr4* sporulating plate, cultured for 7 days, was washed and inoculated in two 250 mL shake flasks containing YEG medium (yeast extract 5 g/L; glucose 20 g/L). As the strain is *pyr4*-deficient, 0.5 M uridine was added. Flasks were incubated at 30 °C in an incubator with 180 rpm for 16–20 h. The culture was transferred to 50 mL tubes and centrifuged (Sigma 212/98, 11,390 Swing-out rotor) at room temperature (RT) with 5000 rpm for 10–20 min to achieve clear supernatant. Pellet was washed 2–3 times with Buffer 1 (1.2 M MgSO_4_; 10 mM Na-phosphate pH 5.8) at RT with 5000 rpm for 10 min. Finally, all the pellets were pulled into single falcon tube and were re-suspended in a 250 mL flask with 90 mL filter sterilized lysing solution (60 mg/mL Vinotaste enzyme or Sigma working concentration of enzymes in Buffer 1 solution). The flask was incubated at 28 °C at 150 rpm until protoplasts were formed (1.5–2.5 h), with monitoring at least 1x per hour. Once the protoplasts were formed, it was split into 3 × 30 mL aliquots in 50 mL conical tubes. Then, 15 mL of Buffer 2 (0.6 M Sorbitol; 0.1 M Tris-HCl pH 7.0) was added carefully as an overlay on top of the filtered protoplast digest suspension at a very slow speed. Tubes were centrifuged at 4500 rpm for 20–30 min with low acceleration and deceleration. Protoplasts were collected form the interphase of the two solutions by positioning the pipette slightly above the interphase and transferred to a new 50 mL falcon tube (approx. 10 mL). Buffer 3 (1.2 M Sorbitol; 10 mM CaCl_2_; 10 mM Tris-HCl pH 7.5) was added to bring the total to 50 mL, which was centrifuged at 4500 rpm for 5 min. After decanting the supernatant, 10 mL of Buffer 3 was added and the pellet was mixed gently by mixing up and down with a sterile 1 mL cut-tip. Again, the volume was brought to 50 mL with Buffer 3 followed by centrifugation. After decanting the supernatant, the protoplasts were re-suspended in 1–5 mL Buffer 3 and cell count/protoplast count was measured using Thoma chamber. The final count was adjusted to 5 × 10^7^–10^8^ protoplasts/mL by adding 0.25 volumes of Buffer 4 (25% PEG 6000; 50 mM CaCl_2_; 10 mM Tris-HCl pH 7.5).

### 2.6. Protoplast Transformation T. reesei

pRPyr4-pc1g plasmid [29] was used to generate prototrophic recombinant strains using RL-P37_Δ*pyr4* as the recipient strain. To 1–10 µg (max. vol. of 20 µL) of purified plasmid in pre-chilled 50 mL falcon tubes, 125–200 µL of protoplasts were added and gently mixed by rotating the tube in clock-wise direction. The mixture was then incubated on ice for 20–30 min. Later, the mixture was brought to RT and 0.8–1 mL of Buffer 4 was added and mixed gently. After 20 min of incubation, 1 mL of Buffer 3 was added and mixed. To this, 25–30 mL of selection medium (MA-agar medium with glucose) was added and the tube was inverted twice before pouring the complete mixture into petri plates. The plates were incubated at 30 °C and colonies appeared from 3 days to 10 days. Colonies were further cultured, keeping the selection pressure, followed by single spore isolation on selection medium with 0.1% IPEGAL (octylphenoxypolyethoxyethanol) or triton-X 100.

### 2.7. RNA Extraction and Analysis of Transcript Levels

Fungal mycelia were homogenized in 1 mL of RNAzol^®^ RT (Sigma-Aldrich, St. Louis, MO, USA) using a FastPrep^®^-24 cell disrupter (MP Biomedicals, Santa Ana, CA, USA) with the intensity 6 for 30 s. RNA was isolated using Direct-zol^TM^ RNA Miniprep kit (Zymo Research, Tustin, CA, USA) according to the manufacturer’s instructions, and the concentration and purity were assessed using a NanoDrop OneC spectrophotometer (Thermo Fisher Scientific, Waltham, MA, USA). Synthesis of cDNA from 500 ng of RNA was carried out using the LunaScript^®^ RT SuperMix Kit (New England Biolabs) according to the manufacturer’s instructions. A QIAgility pipetting robot (QIAGEN, Hilden, Germany) was used for the automated preparation of the qPCR mixes consisting of the Luna Universal qPCR Master Mix (New England Biolabs), 10 μM forward and reverse primer, and 2 μL cDNA (1:50 diluted) as template in a final volume of 15 μL. All reactions were performed in technical duplicates and a no-template control for each primer pair and a no-amplification control (0.015% SDS added to the reaction mixture) was also prepared. Primer sequences are provided in Table 1. The qPCRs were performed in a Rotor-Gene Q system (Qiagen, Hilden, Germany) with version 2.3.1 software. Cycling conditions were the following: 1 min initial denaturation and polymerase activation at 95 °C, followed by 40–45 cycles of denaturation at 95 °C for 15 s and extension at 60 °C for 30 s according to the instructions of the master mix manufacturer. Normalization was performed using *bzp1* as housekeeping gene [31]. The normalized values were related to the reference sample, which is specified in the figure legend, and fold-change values are presented as relative transcript ratios.

### 2.8. Whole Transcriptome Analysis

*T. reesei* GEN-3A was cultivated in fed-batch fermentation using either production medium or minimal medium with glucose in three biological replicates. Samples (in steady-state phase) were collected at 24 h intervals, flash-frozen in liquid nitrogen and stored at −80 °C until RNA extraction. Total RNA was isolated using the Trizol Plus RNA Purification Kit (Life Technologies, Cat. No. 12183555) following the manufacturer’s instructions. Ribosomal RNA was depleted with the NEBNext^®^ RNA Depletion Core Reagent Set in combination with a 50-probe cocktail. Size exclusion purification was performed according to the manufacturer’s guidelines to retain RNA fragments > 200 nt. Libraries were prepared from rRNA-depleted RNA using the NEBNext^®^ Ultra™ II Directional RNA Library Prep Kit for Illumina^®^. RNA was fragmented at 94 °C for 7 min, and PCR enrichment of adapter-ligated cDNA was performed with 10 cycles, starting from 500 ng of input RNA. Libraries were sequenced on an Illumina NextSeq system (300 cycles, paired-end) at a Genencor facility, yielding up to 149 million reads per sample. Raw reads were quality-checked with FastQC (v0.11.9) [32], and trimming of the adapter sequences was performed using fastp (v1.0.1) [33]. Trimmed reads were mapped to the reference RL-P37 genome using HISARL-P37_T2 (v2.2.1) [34]. Transcript assembly was carried out with StringTie (v3.0.1) [35], transcript abundance was quantified with HTSeq (v0.11.1) [36] and differential gene expression analysis was performed using the R package edgeR (v4.6.3) [37]. *p*-values were adjusted for multiple testing using the Benjamini–Hochberg method, and genes were considered differentially expressed at a false discovery rate (FDR) < 0.05. The COG/KOG (Clusters of Orthologous Genes) and GO (Gene Ontology) enrichment analysis was performed by FunFEA, an R package designed for functional enrichment analysis of fungal genomes [38].

## 3. Results

### 3.1. Occurrence of UPR and RESS Under DTT Stress in T. reesei

**Figure 2:**
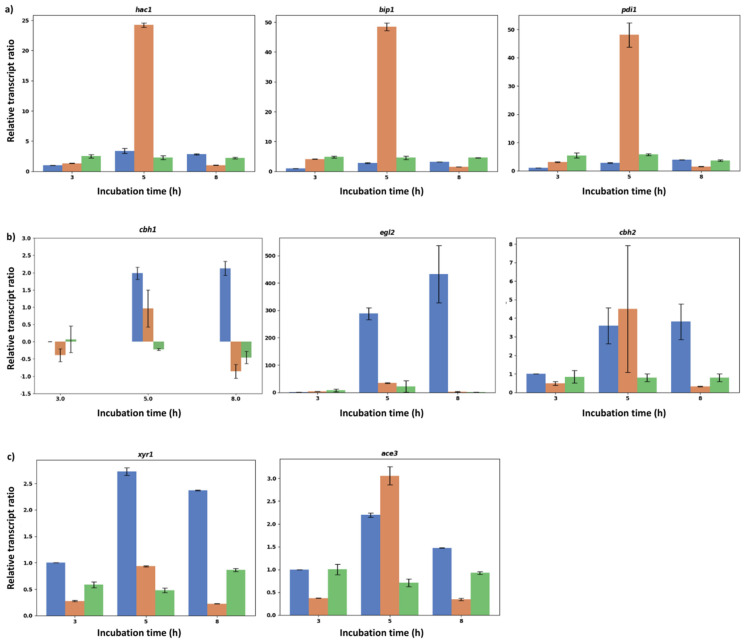
Transcript analysis of *T. reesei* RL-P37 in presence and absence of DTT. Relative transcript levels of (**a**) UPR marker genes (*hac1, bip1,* and *pdi1*), (**b**) RESS target genes (*cbh1*, *egl2*, *cbh2*) and (**c**) transactivator-encoding genes (*xyr1* and *ace3*) without addition of DTT (blue bars), with DTT (orange bars), and with DTT and cycloheximide (green bars) after 3, 5, and 8 h of incubation. Transcript levels were normalized to the housekeeping gene *bzp1*, and the sample without addition of DTT or cycloheximide after 3 h of incubation was used as the reference. Bar plots represent mean values and error bars indicate the standard deviation from technical replicates. In the case of *cbh1*, transcript ratios are given in log-scale.

To assess the activation of the UPR and RESS, *T. reesei* strain RL-P37 was pre-cultivated and transferred to a production medium with DTT, which is used to artificially trigger UPR, and a production medium without DTT. The samples were incubated for 3, 5 and 8 h followed by transcript analysis by RT-qPCR. Transcript levels of UPR-related genes (*hac1*, *bip1*, *pdi1*), cellulase-encoding genes that were reported to be affected by RESS (*cbh1*, *egl2, cbh2*) [25], and transactivator-encoding genes (*xyr1* and *ace3)* were analyzed. The quantitative expression analysis at 5 h revealed a significant upregulation of all three UPR-related genes when DTT was present (Figure 2a, orange bars) compared to the incubation without DTT (Figure 2a, blue bars). As the results demonstrate, this was not the case after 3 h and after 8 h of DTT treatment. This could be explained by the fact that, after 3 h, transcription of these genes was still not induced and, after 8 h, the UPR-related proteins and mechanism could be still on, but the transcription of according genes is no longer activated. The transcripts encoding the RESS target genes were clearly induced in the condition without DTT after 5 and 8 h (Figure 2b, blue bars), and strongly downregulated when DTT was present (Figure 2b, orange bars). The only exception here is the *cbh2* transcript level after 5 h of incubation. However, the strong standard deviation does not allow for any reliable conclusion, while the results for the samples incubated for 3 and 8 h follow the strong reduction pattern as well. Altogether, this supports the presence of the RESS mechanism when DTT is used to artificially trigger UPR. In the case of the transactivators, the transcript level of *xyr1* was reduced whenever DTT was present, while this was not strictly observed for *ace3* (Figure 2c). The reduced *xyr1* transcript levels could potentially be responsible for the reduced levels of the cellulase-encoding genes. However, it needs to be considered that the action of transcription factors is not necessarily dependent on the amount of transcript only, but also on other mechanisms like posttranslational modifications.

### 3.2. UPR, but Not RESS, Can Be Suppressed by Prevention of Protein De Novo Synthesis

To test whether RESS can be prevented by suppressing UPR, cultures were treated with cycloheximide in addition to DTT. Cycloheximide prevents *de novo* synthesis of proteins and, therefore, could interfere with the UPR and/or RESS. Indeed, we only detected basal transcript levels of the UPR-related genes when both DTT and cycloheximide were present (Figure 2a, green bars) comparable to the condition without DTT and cycloheximide (Figure 2a, blue bars), even when transcripts were strongly increased in the presence of DTT only after an incubation of 5 h (Figure 2a, orange bars). This indicates that the addition of cycloheximide prevents the DTT-induced UPR. However, the RESS target genes *cbh1, egl2* and *cbh2* were at similarly low levels when incubated with DTT and cycloheximide (Figure 2b, green bars) when incubated with DTT only (Figure 2a, orange bars). Conclusively, RESS was not prevented by cycloheximide addition, whereas UPR was inhibited. In the case of the transactivators, again, *xyr1* transcript levels were always reduced in the presence of DTT and cycloheximide compared to the condition without the two reagents. This could indirectly influence the transcript levels of cellulase-encoding genes. Otherwise, *ace3* transcript levels were not always reduced (Figure 2c).

### 3.3. RESS Acts on the Level of Transcription Initiation

**Figure 3:**
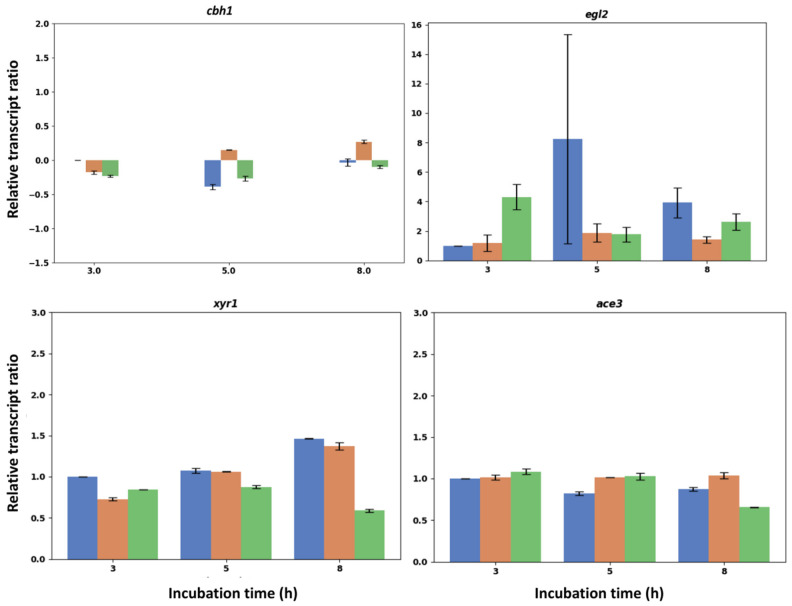
Transcript analysis of *T. reesei* RL-P37 in presence and absence of DRB and DTT. Relative transcript levels of *cbh1*, *egl2*, *xyr1* and *ace3* after incubation in production medium (blue bars), in the presence of DRB (orange bars) and in presence of DRB and DTT (green bars) after 3, 5, and 8 h of incubation. Transcript levels were normalized to the housekeeping gene *bzp1*, and the RL-P37 without addition of DRB or DTT after 3 h of incubation was used as the reference. Bar plots represent mean values and error bars indicate the standard deviation from technical replicates. In the case of *cbh1*, transcript ratios are given in log-scale.

As the presence of RESS could be verified by the above-described experiments, we wanted to know whether this mechanism acts at the level of the transcription and/or influences the degradation of mRNA. The hypothesis for designing the next experiment was the following: if an inhibitor of transcription, for example, DRB, which is a Pol II elongation inhibitor, is present, a decrease in any transcript of interest can be expected. Therefore, an incubation of the fungus in the presence of both DRB and DTT can lead to two likely results: either the same reduction in transcript levels is observed as with DRB only, which would suggest that RESS acts on the level of transcription only, or a stronger reduction in transcript levels is observed, which would mean that mRNA degradation alone or in combination with the level of transcription are the RESS-related mechanisms. Accordingly, *T. reesei* was pre-cultivated and mycelium was transferred to the production medium, production medium with DRB, and production medium with DRB and DTT. Incubation was performed for 3, 5 and 8 h followed by transcript analysis of RESS target genes *cbh1* and *egl2* as well as the transactivator-encoding genes *xyr1* and *ace3* by RT-qPCR. An overall conclusive result could not be obtained. While the *cbh1* transcript after 3 h of incubation was reduced by the addition of DRB (Figure 3, orange bars) compared to the sample without DRB (Figure 3, blue bars) as expected, at the other two time points, the transcript level was higher when DRB was present. In the case of *egl2*, transcript levels were similar (3 h) or reduced (5 and 8 h) in the presence of DRB (Figure 3, orange bars) compared to without DRB (Figure 3, blue bars); the latter two are as expected. For the following interpretation of results, we only considered the time points for which the DRB control gave the expected reduced transcript levels, i.e., 3 h in the case of *cbh1* and 5 and 8 h in the case of *egl2*. The addition of DRB and DTT led to a similar *cbh1* transcript level (Figure 3, green bars) compared to DRB only after 3 h of incubation (Figure 3, orange bars), which would point to the action of RESS on the transcription level only. Also, the transcript levels of *egl2* were similar (5 and 8 h) in the presence of DRB and DTT (Figure 3, green bars) compared to DRB only (Figure 3, orange bars), which would again point to the transcription initiation as the mechanism on which RESS acts. In the case of the transactivator-encoding genes, transcript levels remained similar or were even slightly higher in the presence of DRB (Figure 3, orange bars) compared to the condition without DRB (Figure 3, blue bars) except for the *xyr1* transcript after 3 h of incubation. Again, the additional usage of DTT did reduce *xyr1* transcript levels, but not strictly the *ace3* transcript levels (Figure 3, green bars). Neither *xyr1* nor *ace3* transcript levels follow the same pattern as the *cbh1* or *egl2* transcript levels. This does not support a sole indirect effect of the transcript abundance of the transactivator-encoding genes on the RESS target genes.

However, we decided to investigate the possible RESS mechanisms additionally with the usage of recombinant strains.

For this purpose, three recombinant *T. reesei* strains (RL-P37_T1, RL-P37_T2 and RL-P37_T3) were generated via homologous recombination using RL-P37_Δ*pyr4* as recipient strain and restoration of the *pyr4* locus as the selection marker (Figure 1). The RESS target gene, *cbh1*, was one time put under the promotor and terminator of the *gpd1* gene, which is not an RESS target gene, to achieve a release from the two most likely target sequences of RESS. In a second strain, the terminator of *gpd1* was used instead of the *cbh1* terminator to see whether the terminator sequence is a target of RESS and, for example, affects mRNA stability. And, in a third strain, the promoter of *gpd1* drove the expression of the *cbh1* gene, while, as terminator, the one from another RESS target gene, namely *cbh2*, was used to see whether RESS acts exclusively on the level of transcription initiation. The genotype of these strains was confirmed by PCR and subsequent sequencing.

**Figure 4:**
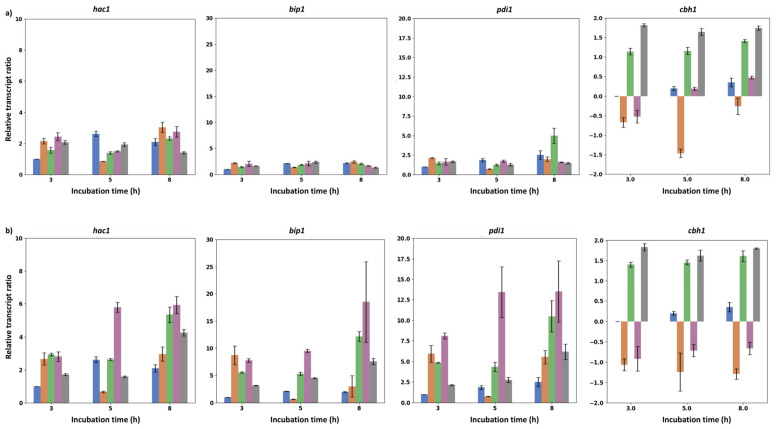
Transcript analysis of recombinant *T. reesei* RL-P37 and recombinant strains. Relative transcript levels of UPR-related genes (*hac1*, *bip1*, and *pdi1*) and *cbh1* without addition of DTT (**a**) and with DTT (**b**) after 3, 5, and 8 h of incubation. (**a**) RL-P37 (blue bars) as the reference strain, the parent strain RL-P37_Δ*pyr4* (orange bars) as control, and recombinant strains RL-P37_T1 (promoter and terminator exchange; green bars), RL-P37_T2 (terminator exchange; purple bars) and RL-P37_T3 (promoter exchange; grey bars). (**b**) RL-P37 without DTT (blue bars) as the reference condition, RL-P37 (orange bars with dots), and recombinant strains RL-P37_T1 (promoter and terminator exchange; green bars), RL-P37_T2 (terminator exchange; purple bars) and RL-P37_T3 (promoter exchange; grey bars). Transcript levels were normalized to the housekeeping gene *bzp1*, and the RL-P37 without addition of DTT after 3 h of incubation was used as the reference. Bar plots represent mean values and error bars indicate the standard deviation from technical replicates. In the case of *cbh1*, transcript ratios are given in log-scale.

For clarifying the above-mentioned possibilities, RL-P37, the three recombinant strains and their parent strain RL-P37_Δ*pyr4* were pre-cultivated and transferred to production medium with and without the addition of DTT and incubated for 3, 5, and 8 h. Then, transcript analysis of UPR-related genes (*hac1*, *bip1*, *pdi1*) and the RESS target gene *cbh1* was performed by RT-qPCR. Without the addition of DTT, the UPR-related genes were expressed at similar low levels in all investigated strains and time points (Figure 4a). For the target gene *cbh1*, a slight increase over time was observed in RL-P37. Interestingly, *cbh1* transcript levels were clearly higher in the strain with promoter and terminator exchange and were even more strongly increased in the strain with promoter exchange (Figure 4a). This points to a more favorable regulatory environment by usage of a different promoter (and terminator) than for the native *cbh1* promoter/terminator.

**Figure 5:**
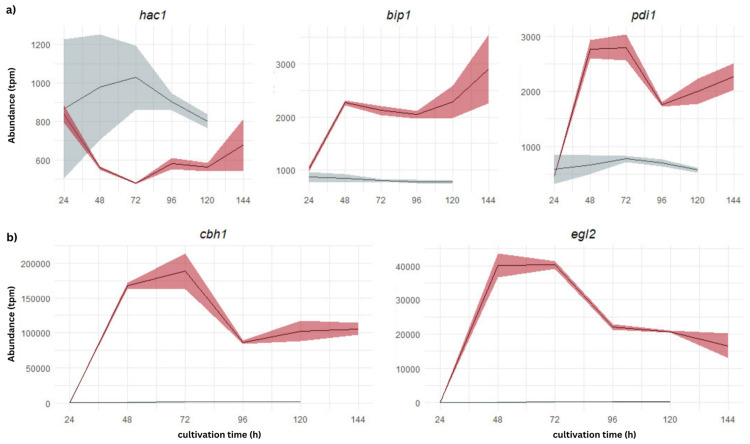
Transcript abundance profiles of *Trichoderma reesei* GEN-3A during bioreactor cultivation. Whole transcriptome sequencing analysis was performed on samples collected after 24, 48, 72, 96, 120, and 144 h of cultivation under cellulase-inducing conditions (cellulase production medium; red lines) and control conditions (glucose; grey lines). Transcript abundance profiles are shown for UPR-related genes (*hac1*, *pdi1*, and *bip1*) (**a**) and RESS target genes (*cbh1* and *egl1*) (**b**). Lines represent mean transcript abundances across biological triplicates, and shaded areas indicate standard deviation.

When DTT was added, the transcript levels of the investigated UPR-related genes were increased in all strains after incubation for 3 and 8 h and partly after incubation for 5 h compared to the incubation without DTT (Figure 5b, RL-P37 without DTT is again displayed for better comparison). This confirms that UPR was triggered by the addition of DTT. The observed slight expression of *cbh1* in RL-P37 without DTT (Figure 5b, blue bars) was lost upon the addition of DTT (Figure 4b, orange bars), confirming the earlier-described RESS mechanism acting on *cbh1*. Accordingly, the detected low *cbh1* transcript levels in the strain with exchanged terminator (Figure 4a, purple bars) were lost upon the addition of DTT (Figure 4b, purple bars). As the exchange of the terminator only did not lead to increased transcript levels, this indicates that the *cbh1* terminator is not the target on which RESS acts. On the opposite side, it seems that the usage of this terminator supported the degradation of *cbh1* mRNA. On the other hand, in both recombinant strains with the promoter exchanged, the *cbh1* transcript levels were again strongly elevated (Figure 4b) and were even more highly increased in the presence of DTT (Figure 4b) than in the absence of DTT (Figure 4a). This suggests that RESS acts on the level of transcription initiation.

### 3.4. Whole Transcriptome Analysis Reveals Distinct Expression Patterns of UPR-Related and Cellulase-Encoding Genes

Besides the usage of DTT to trigger UPR in lab-scale experiments, we were interested in what the transcript profiles of UPR-related genes and RESS target genes look like in actual industrial cellulase production conditions. The industrial *T. reesei* strain GEN-3A was cultivated in bioreactors on a production medium for cellulase expression and on glucose as a control condition. Samples were collected after 24, 48, 72, 96, 120, and 144 h of cultivation and whole transcriptome analysis was performed.

In the cellulase production condition, we detected a rise in transcript abundance of the UPR-related genes *bip1* and *pdi1*, which was not observed in the control condition (Figure 5a). In the case of the UPR-related *hac1*, we observed a different trend (first a decrease and later a rise under the cellulase production condition) (Figure 5a), which could be attributed to the fact that the transcript levels of a transcription factor develop differently than the ones of proteins directly involved in the UPR. Altogether, we assumed that, after 48 h of cultivation under cellulase production conditions, UPR is on. The transcript abundance of the two target genes *cbh1* and *egl1* peaked from 48 to 72 h under the cellulase production condition, followed by a decline and rather stable expression after 96 h of cultivation (Figure 5b). For *cbh1*, we detected a very high transcript abundance (approaching 200,000 normalized counts), whereas *egl2* reached lower maximal levels (~50,000). As expected, hardly any transcripts of *cbh1* and *egl1* could be detected on glucose due to carbon catabolite repression (Figure 5b). Importantly, neither *cbh1* nor *egl2* exhibited the characteristic of RESS, indicating sustained cellulase gene expression under inducing conditions.

**Figure 6:**
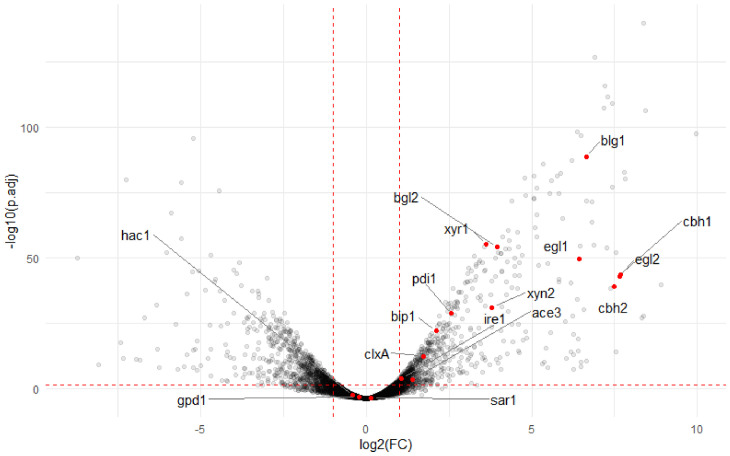
Volcano plot of differential gene expression in *Trichoderma reesei* GEN-3A after 72 h of bioreactor cultivation. Each point represents a gene, plotted by log2 fold change between cellulase production medium and glucose control conditions on the *x*-axis and statistical significance on the *y*-axis (−log10-adjusted *p*-value; Bonferroni correction), and the horizontal red line indicates the significance threshold (*p* < 0.05). Genes of interest are highlighted in red and include secreted cellulolytic enzymes (*cbh1*, *cbh2*, *egl1*, *egl2*, *xyn2*, *bgl1*, and *bgl2*), key transcriptional regulators of cellulase expression (*xyr1* and *ace3*), UPR-related genes (*hac1*, *bip1*, *pdi1*, *ire1*, and *clxA*), and selected non-UPR genes (*gpd1* and *sar1*).

Since the latter was a surprising result, we analyzed in detail the whole transcriptome at 72 h of cultivation because this was the time point at which *cbh1*, *egl1*, and *pdi1* transcripts peaked, and also *bip1* was clearly expressed. According to the Volcano plot provided in Figure 6, the expression of the RESS target genes *cbh1* and *egl2* along with additional hydrolytic enzymes such as *xyn2*, *egl1*, *blg1* or *cbh2* was found to be in the upper right quadrant, indicating a strong differential expression when comparing the cellulase production condition to the control condition and a high degree of statistical significance. While the UPR-related transcription-factor-encoding gene *hac1* remained below the threshold line, other UPR-related genes, in particular, *bip1* and *pdi1,* were found to be differentially upregulated (Figure 6). This, again, supports that, even though UPR was active, RESS could not be detected.

**Figure 7:**
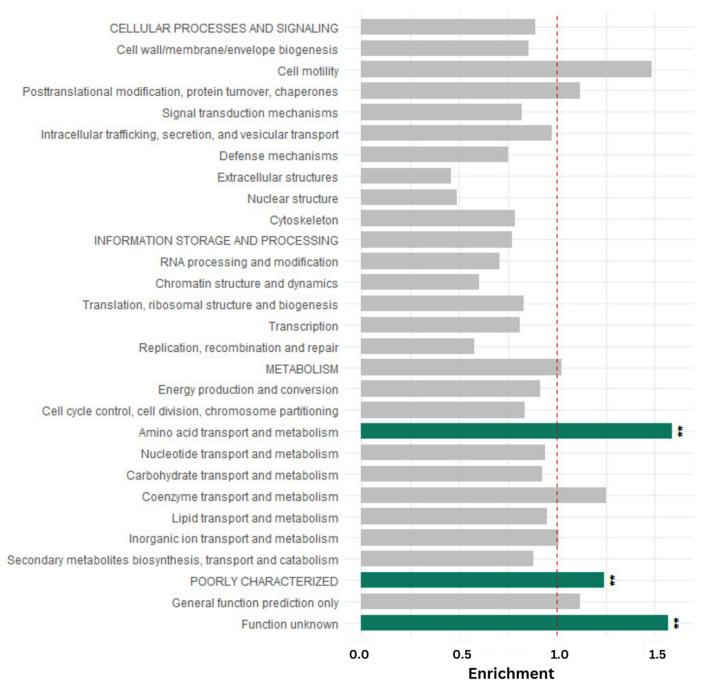
KOG functional enrichment analysis of differentially expressed genes in *Trichoderma reesei* GEN-3A after 72 h of bioreactor cultivation. Differentially expressed genes identified at the 72 h time point were assigned to KOG functional categories, and enrichment was calculated relative to the genomic background. Bars represent enrichment values for each KOG functional category. Bars are colored to indicate statistically significant enrichment. The red dotted line indicates an enrichment value of 1, corresponding to no enrichment relative to background expectation. Statistical significance of enrichment is indicated by asterisks (** = *p* < 0.01).

Functional annotation and classification of differentially expressed genes were also performed using the KOG database. KOG functional enrichment analysis using FunFEA [38] classified the gene set into major biological categories, highlighting distinct trends in cellular function. Among the broad category of cellular processes and signaling, subcategories such as cell motility and posttranslational modification, protein turnover, and chaperones were found to be enriched (Figure 7). Metabolism displayed a pronounced enrichment; in particular, the subcategories amino acid transport and metabolism were significantly above the threshold. The poorly characterized group also exhibited significant enrichment, including both function unknown and general function prediction only subcategories (Figure 7).

**Figure 8:**
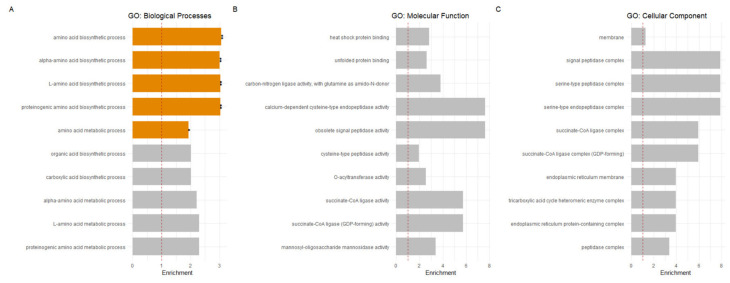
Gene Ontology (GO) enrichment analysis of differentially expressed genes in *Trichoderma reesei* GEN-3A after 72 h of bioreactor cultivation. Differentially expressed genes identified at the 72 h time point were assigned to GO terms and analyzed separately for (**a**) biological processes, (**b**) molecular function, and (**c**) cellular component categories. Enrichment was calculated relative to the genomic background. Bars represent enrichment values for each GO term. Bars are colored to indicate statistically significant enrichment. The red dotted line indicates an enrichment value of 1, corresponding to no enrichment relative to background expectation. Statistical significance of enrichment is indicated by asterisks (* = *p* < 0.05; ** = *p* < 0.01).

The GO analysis using FunFEA v1.2.2 [38] provides a comprehensive functional annotation of gene sets, categorizing them into biological processes, molecular functions, and cellular components. The GO analysis provided in Figure 8 visualizes the significantly enriched GO terms among the differentially expressed genes identified in the sample collected after 72 h. In the biological processes group, five categories related to amino acid biosynthesis were highly enriched and statistically robust, exceeding the enrichment threshold indicated by the dotted line. The remaining categories in this group, shown in grey, did not reach the same significance level (Figure 8a). Within the molecular function group, two categories, i.e., calcium-dependent cysteine-type endopeptidase activity and obsolete signal peptidase activity, surpassed the enrichment threshold; however, they were not at the highest statistical confidence (Figure 8b). In the cellular component group, three categories related to peptidase complex showed enrichment above the threshold, without reaching the stringent significance criteria (Figure 8c).

## 4. Discussion

### 4.1. Persistence of RESS Despite Translation Inhibition

Our findings are in accordance with the earlier-reported results on strain Rut-C30, where an increase in the transcript levels of UPR-related genes indicated that UPR can be triggered by the addition of DTT [25,39]. Also, we observed a downregulation in the transcript level of genes encoding secretory proteins (*cbh1* and *egl2)* in the presence of DTT, which was earlier described as the unique mechanism RESS [25]. However, the literature evidence regarding the corelation of UPR and RESS was rather meagre. Furthermore, the reversal of RESS by the addition of cycloheximide was reported previously for Rut-C30 [25]. Cycloheximide is a well-established protein translation inhibitor that acts by blocking the elongation phase of eukaryotic translation. It binds to the ribosome and inhibits the eEF2-mediated translocation step, effectively halting new protein synthesis in eukaryotic cells [40]. Therefore, it can be expected that such an artificial prevention of protein *de novo* synthesis suppresses the UPR. And, indeed, we found, in our study, that transcript levels of the investigated UPR-related genes were at similarly low levels in the presence of DTT and cycloheximide to in a medium without them, while they were increased in the presence of DTT only, which suggests that cycloheximide did prevent UPR. Another unexpected result was finding the target genes *cbh1* and *egl2* to be still on a repressed level. This would suggest that DTT can trigger RESS but not via UPR.

### 4.2. Potential Mechanisms of RESS

To gain insight into the mechanism of RESS, we examined transcript levels of the RESS-target genes *cbh1* and *egl2* following transcription inhibition by DRB in the presence and absence of UPR-mediated induction by DTT. At the time points at which we detected the expected decrease in transcript levels upon the addition of DRB, the levels remained similar if DTT, in addition to DRB, was added to the medium. This suggested an action of RESS on the level of transcription rather than via the involvement of mRNA degradation.

In a follow-up experiment, recombinant strains were created wherein the promoter (testing whether RESS acts at the level of transcription initiation), the terminator (testing whether RESS acts via mRNA degradation) or both of the RESS target genes *cbh1* were exchanged with the one from the non-UPR target gene *gpd1.* The *gpd1* promoter is widely used as a strong constitutive promoter for heterologous gene expression in *T. reesei* [41] and is not directly affected by UPR [42].

Our findings demonstrate that the substitution of the *cbh1* promoter with that of the *gpd1* gene effectively prevented RESS. The *cbh1* transcript levels in both strains bearing the exchanged promoter (T1 and T3) were the same or higher in the presence of UPR triggered by DTT (compare Figure 5a,b). Interestingly, transcript levels of these two strains were strongly increased compared to the parent strain, which could be attributed to a generally stronger promoter and/or favorable regulatory conditions like the lack of Cre1 binding sites and with that of carbon catabolite repression. We also observed that the strain with the promotor-only exchange clearly performed better than the strain with promoter and terminator exchanged. Obviously, the terminator exchange interfered with the strongly increased transcript formation caused by the promoter exchange. This could be explained by increased mRNA instability. Besides this, we also observed a stronger UPR indicated by higher related transcripts in the strain bearing promoter and terminator compared to the strain bearing only the promoter exchange (see Figure 5b). Notably, the strain that only had the terminator exchanged had the strongest UPR according to the transcript levels of according genes. In the same strain, almost no *cbh1* transcript was detected, even less than in the parent strain. Again, this could point to *cbh1* mRNA being more prone to degradation because of the exchanged terminator.

An earlier study systematically evaluated combinations of various promoters and terminators in fungal gene expression [43]. They found that, while both promoters and terminators can modulate gene expression, the promoter had a predominant effect on transcript abundance. When both elements (promoter and terminator) were exchanged, the gene expression level largely resembled that obtained with the promoter exchange alone. The authors concluded that promoters and terminators could be applied independently to tune gene expression, but the promoter typically drives the major regulatory outcome [43]. Our study supports the superior impact of promoter elements. In this case, the removal of promoter elements resulted in a release of *cbh1* gene expression from RESS. The terminator exchange, by contrast, was not only unable to suppress RESS, but it even led to a further reduction in *cbh1* transcript levels.

### 4.3. Absence of Classical UPR and RESS Responses in T. reesei at Industrial Scale

While cellulase induction is well established, the interplay between stress responses and cellulase production under industrially relevant conditions remains incompletely characterized [20,44]. A whole transcriptome analysis of an industrial cellulase production process revealed that the transcript abundance of key cellulase genes (*cbh1*, *egl2*) and UPR-related genes (*pdi1*, *bip1*) peaked between 48 and 72 h under cellulase-inducing conditions.

In this context, we made two interesting observations: first, *hac1* transcript formation was not found to be increased as *pdi1* and *bip1* genes. This could align with previous reports indicating that mild-to-moderate secretory load during efficient cellulase synthesis does not necessarily trigger canonical UPR activation. Meanwhile, the elevated expression of chaperones such as *pdi1* and *bip1* appears to be linked to increased protein folding demand due to carbon source induction rather than cellular stress *per se* [6]. Their temporal expression pattern showing a strong early peak followed by decline may reflect substrate availability and metabolic adaptation over the fermentation course [45]. For example, *pdi1* expression was reported to be influenced by the carbon source, with higher mRNA levels observed in media containing cellulase-inducing substrates (e.g., lactose, sophorose) compared to glucose. This regulation is mediated by carbon catabolite repression via the Cre1 protein, as demonstrated by elevated *pdi1* expression in the *cre1* mutant strain Rut-C30 on glucose [46]. *Bip1* transcript levels correlate with secretory demands during growth phases. For example, in chemostat cultures, both *bip1* and *pdi1* are upregulated at low growth rates (≈0.03 h^−1^) to optimize protein folding capacity for efficient cellulase secretion, independent of ER stress [47]. Another piece of evidence brings forward that overexpression of the transactivator Xyr1, which is essential for cellulase production, does not induce UPR markers (*hac1*, *bip1*), suggesting that secretory machinery components like Bip1 and Pdi1 may be regulated directly by secretion-related signals rather than ER stress [48,49].

Second, the maintenance of high *cbh1* and *egl2* transcript levels contrasts with an RESS response, where cellulase gene expression is expected to be downregulated. This suggests that the production process was primarily regulated by carbon source signals without triggering deleterious secretion feedback mechanisms, which is consistent with effective industrial enzyme production strategies. In accordance, the KOG enrichment plot highlights the processes under the metabolism category and suggests a pronounced shift in metabolic activity, likely reflecting adaptive responses for substrate utilization or biosynthetic requirements under the experimental conditions [50].

As a final point, it should be acknowledged that experiments were performed as time course, but not in biological replicates. Consequently, several of the reported results should be interpreted as trends rather than definitive conclusions. As a future research direction, more biological replicates would allow for a stronger statistical validation of some of the presented findings. Also, the analysis of the UPR and the RESS in the context of heterologously expressed proteins in *T. reesei* would expand the so-far-obtained knowledge on these two mechanisms.

## 5. Conclusions

This study demonstrates that the UPR and RESS can be artificially induced by DTT in *Trichoderma reesei*. However, there are a number of findings that would point to a non-hierarchical connection between these two mechanisms. The prevention of protein *de novo* synthesis did suppress UPR as expected, but it did not interfere with RESS. Also, recombinant strains with an exchanged promoter had a higher UPR than their parent strain, and, simultaneously, a release from RESS. Whether DTT and RESS are connected or co-regulated remains open. The mentioned promoter exchange experiments demonstrated that RESS acts on the level of transcription initiation, offering an obvious target for strain improvement. On the contrary, terminator exchange had the opposite effect.

While a reductive chemical induction under laboratory conditions triggered UPR and RESS, the absence of these signatures during industrial cellulase production suggests that these stress responses may be modulated or attenuated in real protein load environments. It should be noted that stress signaling pathways characterized in artificial lab settings may not fully reflect physiological stress regulation during large-scale bioprocesses.

## Figures and Tables

**Table 1:** List of primers used throughout this study.

Primer Name	Sequence (5′ ⟶ 3′)
Plasmid construction	
Backbone1_fwd	CAATGAGGTCTTGGATGGCTTTCCAAAC
Backbone1_rev	GTCAGTCGCACCCGGAGTAGCTCTTCAC
P*gpd1*_fwd	CTACTCCGGGTGCGACTGACATTCGACC
P*gpd1*_rev	GCGCAGTCCGTCGTCTGTGCCGCCAATG
*cbh1*_fwd	GCACAGACGACGGACTGCGCATCATGTATC
*cbh1*_rev	CCAATCCAACTTACAGGCACTGAGAGTAGTAAG
T*gpd1*_fwd	GTGCCTGTAAGTTGGATTGGTAATAAAGGCTTTTAGGC
T*gpd1*_rev	AGCCATCCAAGACCTCATTGCCGAGCGC
Backbone2_fwd	CAATGAGGTCTTGGATGGCTTTCCAAACGTTAATAG
Backbone2_rev	GCGCAGTCCGGGATCCGATGCGCAGTCC
*cbh1*_fwd	CATCGGATCCCGGACTGCGCATCATGTATC
*cbh1*_rev	CCAATCCAACTTACAGGCACTGAGAGTAGTAAG
T*gpd1*_fwd	GTGCCTGTAAGTTGGATTGGTAATAAAGGCTTTTAGGC
T*gpd1*_rev	AGCCATCCAAGACCTCATTGCCGAGCGC
Backbone3_fwd	GTGCCTGTAAATTCTGGATCCTTTCGTGAC
Backbone3_rev	GTCAGTCGCACCCGGAGTAGCTCTTCAC
P*gpd1*_fwd	CTACTCCGGGTGCGACTGACATTCGACC
P*gpd1*_rev	GCGCAGTCCGTCGTCTGTGCCGCCAATG
*cbh1*_fwd	GCACAGACGACGGACTGCGCATCATGTATC
*cbh1*_rev	GATCCAGAATTTACAGGCACTGAGAGTAGTAAG
Genotype verification	
p*gpd1*_f	TTCGACCTTCCTGGATTGCC
p*gpd1*_r	CCTGGTAGACTTGGGGGAGA
*cbh1*_F	TACGAACAGCAGCACGAACT
*cbh1*_R	AGAGCCTCGGAGATGGAGTT
t*gpd1*_f	GCTATTACCCGGGGCTTCTC
t*gpd1*_r	TTCTGACTCCCCGAGCCATA
qPCR	
Rj_Hac1_F	AACCTCCCTCCTCGAAAACG
Rj_Hac1_R	AGGATCAGGTTGGTCTTCTG
Rj_Cbh1_F	ACTATGTCCAGAATGGCGTC
Rj_Cbh1_R	TGGCGTAGTAATCATCCC
Rj_*egl2*_F	CCACTACTATCACCACTTCG
Rj_*egl2*_R	ACGCAAGTGCCATCTGTG
*pdi1*f	AATGACCTCGTCCTGGCTGA
*pdi1*r	GGCGAGCTTGATGCTCTTGT
*bip1*f	GAGGGTGAGCGTTCCATGAC
*bip1*r	GTTGGCATCCAACTCGAAGG
*bzp1*f	GGCCTTTCTTTGAGCAGTGATG
*bzp1*r	AGCTGCCCTTTGTTGTTGTC

## Data Availability

The data presented in this study are available on request from the corresponding author due to reasons of confidentiality.
